# Pathogenic mitochondrial mt-tRNA^Ala^ variants are uniquely associated with isolated myopathy

**DOI:** 10.1038/ejhg.2015.73

**Published:** 2015-04-15

**Authors:** Diana Lehmann, Kathrin Schubert, Pushpa R Joshi, Steven A Hardy, Helen A L Tuppen, Karen Baty, Emma L Blakely, Christian Bamberg, Stephan Zierz, Marcus Deschauer, Robert W Taylor

**Affiliations:** 1 Department of Neurology, University of Halle-Wittenberg, Halle (Saale), Germany; 2 Wellcome Trust Centre for Mitochondrial Research, Institute of Neuroscience, The Medical School, Newcastle University, Framlington Place, Newcastle upon Tyne, UK; 3 Department of Neurology, Rhein-Mosel-Fachklinik, Andernach, Germany

## Abstract

Pathogenic mitochondrial DNA (mtDNA) point mutations are associated with a wide range of clinical phenotypes, often involving multiple organ systems. We report two patients with isolated myopathy owing to novel mt-tRNA^Ala^ variants. Muscle biopsy revealed extensive histopathological findings including cytochrome *c* oxidase (COX)-deficient fibres. Pyrosequencing confirmed mtDNA heteroplasmy for both mutations (m.5631G>A and m.5610G>A) whilst single-muscle fibre segregation studies (revealing statistically significant higher mutation loads in COX-deficient fibres than in COX-positive fibres), hierarchical mutation segregation within patient tissues and decreased steady-state mt-tRNA^Ala^ levels all provide compelling evidence of pathogenicity. Interestingly, both patients showed very high-mutation levels in all tissues, inferring that the threshold for impairment of oxidative phosphorylation, as evidenced by COX deficiency, appears to be extremely high for these mt-tRNA^Ala^ variants. Previously described mt-tRNA^Ala^ mutations are also associated with a pure myopathic phenotype and demonstrate very high mtDNA heteroplasmy thresholds, inferring at least some genotype:phenotype correlation for mutations within this particular mt-tRNA gene.

## Introduction

Mitochondrial DNA (mtDNA) disorders are associated with a wide range of different clinical phenotypes, from mild to severe.^1^ Mutations affecting mitochondrial (mt)-tRNA genes are prevalent amongst adults and usually associated with multisystemic disease presentations;^2^ isolated organ involvement is rarely observed. Furthermore, it is unusual for mutations in one specific mt-tRNA gene to associate with a unique clinical phenotype, although several mutations in mt-tRNA^Leu(UUR)^ and mt-tRNA^Ile^ are linked to MELAS and mitochondrial cardiomyopathy respectively.^3^

The overwhelming majority of pathogenic mtDNA mutations are present in a heteroplasmic state, the level of mtDNA mutation within a cell or tissue required to exceed a critical threshold to cause a disease phenotype.^4^ This threshold level varies for each mutation and tissue and is dependant on several factors including OXPHOS metabolism.^5, 6^ Here we report two patients, both presented with isolated myopathy, with novel heteroplasmic mutations in the mt-tRNA^Ala^ gene exhibiting high thresholds for disease expression.

## Patients and methods

### Patient 1

Patient 1 is a young lady who presented at the age of 29 years with muscle weakness, initially involving her hands but spreading to her arms and legs progressively, resulting in her being non-ambulatory by the age of 40 years. Her mother is healthy with no history of muscle disease, whereas the patient has no children or siblings. She was noted to have a mild symmetrical ptosis but with no history of myoglobinuria, diabetes or seizures. Neurological examination revealed general floppy tetraparesis (MRC 3-4/5). Muscle tendon reflexes were weak. Sensory examination results, tone and Babinski reflexes were all normal. Electroencephalogram revealed no signs of increased cerebral excitability. Needle electromyogram of the biceps brachii muscle and the vastus medialis muscle revealed distinctive myopathic changes. Nerve conduction studies of the suralis nerve showed normal results. Thigh-MRI showed distinctive fatty degeneration of all muscle groups, equal on both sides. Cerebral MRI, cardiac MRI and audiogram were normal. Creatine kinase was slightly elevated (3.9 *μ*mol/l; normal: <2.4 *μ*mol/l). Resting lactate level was normal (2.0 mmol/l; normal: <2.8 mmol/l).

### Patient 2

This lady presented at the age of 69 years with progressive weakness of the limb girdle muscles including proximal paresis. There is no known muscle disease or muscle weakness in the family. Ptosis, ophthalmoplegia or extramuscular mitochondrial symptoms have not been noticed. Neurological examination revealed dysarthria, a facies myopathica and proximal paresis (MRC 3-4/5). Sensory examination and Babinski reflexes were normal. Muscle tendon reflexes on the upper extremity were weak. Achilles deep-tendon reflexes were not obtainable on both sides. Patella deep-tendon reflexes were left accentuated obtainable. Needle electromyogram of the deltoideus muscle and the biceps brachii muscle revealed myopathic changes. Creatine kinase was slightly elevated (2.7 *μ*mol/l; normal: <2.4 *μ*mol/l), as was the resting lactate level (2.9 mmol/l; normal: <2.8 mmol/l).

### Histopathology and molecular genetic studies

Standard histopathologic analysis of muscle biopsies of both patients was performed and the activities of respiratory chain complexes were determined spectrophotometrically.^7^ Total DNA from all available tissue (muscle, urinary epithelia, buccal epithelia, hair shafts and blood), including tissue from available maternal relatives, was extracted by standard procedures. Long-range PCR of muscle DNA was undertaken to detect large-scale mtDNA rearrangements,^8^ followed by sequencing of the entire mitochondrial genome in this tissue.^9, 10^ Analysis of mtDNA heteroplasmy was carried out by quantitative pyrosequencing including segregation studies within individual cytochrome *c* oxidase (COX)-positive and COX-deficient fibres.^11^ High-resolution northern blotting to assess mt-tRNA^Ala^ steady-state levels in both patients was performed as described.^12^

## Results

**Figure 1:**
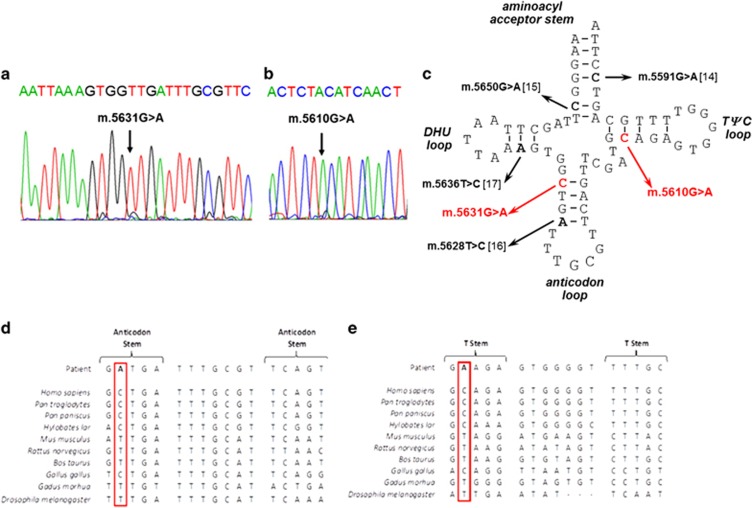
Identification of novel mt-tRNA^Ala^ variants (**a**) Sequencing electropherogram (reverse sequence) demonstrating the heteroplasmic m.5631G>A transition detected in patient muscle. (**b**) Sequencing electropherogram demonstrating the heteroplasmic m.5610G>A transition detected in muscle. (**c**) Schematic representation of the mt-RNA^Ala^ cloverleaf structure, illustrating the position of the novel m.5631G>A and m.5610G>A variants and other reported mt-tRNA^Ala^ mutations. Phylogenetic conservation of the appropriate regions of the mt-tRNA^Ala^ gene sequence for both (**d**) m.5631G>A and (**e**) m.5610G>A indicates that both variants affect an evolutionary conserved residue.

**Figure 2:**
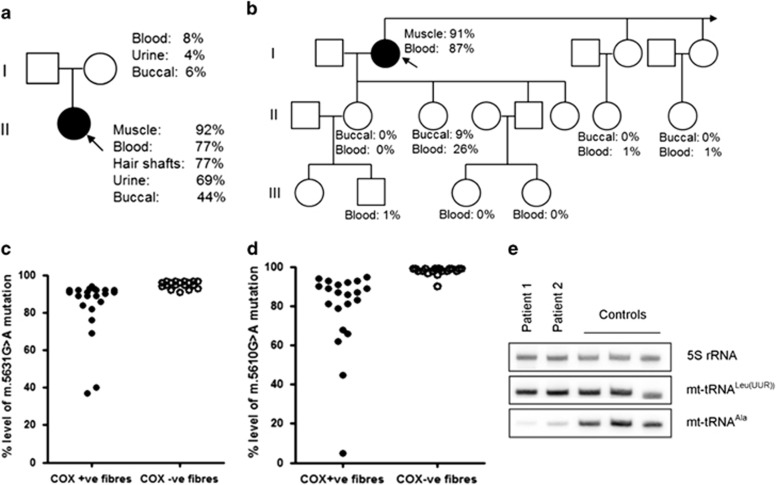
Molecular genetic investigations of patients' muscle with the novel m.5631G>A and m.5610G>A variants (**a**) Patient 1's pedigree including mtDNA mutation heteroplasmy levels in the index case and her mother. (**b**) Family pedigree including mtDNA mutation heteroplasmy levels in the index case (Patient 2) and other family members. (**c**) Single-fibre PCR analysis of the m.5631G>A mutation segregates with a biochemical defect in individual COX-deficient muscle fibres. (**d**) Single-fibre PCR analysis of the m.5610G>A mutation segregates with a biochemical defect in individual COX-deficient muscle fibres. (**e**) Measurement of mt-tRNA^Ala^ steady-state levels in muscle showing dramatically decreased mt-tRNA^Ala^ steady-state levels in both patients.

Muscle biopsy analysis revealed numerous COX-deficient (Patient 1: 33%, Patient 2: 40%) and ragged-red-fibres (Patient 1: 2%, Patient 2: 5%) in both patients. Biochemical analysis of muscle from Patient 1 showed decreased activities of respiratory chain complexes I, II/III and IV, and a compensatory increase in citrate synthase activity. Decreased activities of respiratory chain complexes I and IV, with a compensatory increase in citrate synthase activity, were noted in Patient 2. Long-range PCR failed to identify large-scale mtDNA deletions, prompting sequencing of the entire mitochondrial genome in muscle which revealed novel mt-tRNA^Ala^ (*MTTA*) gene mutations – m.5631G>A (ClinVar Reference SCV000196082: NC_012920.1) in Patient 1 and m.5610G>A (ClinVar Reference SCV000196083: NC_012920.1) in Patient 2 (Figure 1c). The highest m.5631G>A mutation load in Patient 1 was found in muscle (92% levels of mtDNA heteroplasmy), with lower levels present in blood (77%), hair shafts (77%), urinary epithelial sediment (69%) and buccal epithelial cells (44%). In Patient 2, the highest mutant load was detected also in muscle (91% levels of mtDNA heteroplasmy), followed by blood (87%). Levels of mtDNA heteroplasmy detected in additional family members are shown (Figures 2a and b).

Single-muscle fibre analysis of individual COX-positive and COX-deficient fibres of both cases revealed a statistically significant higher mutation load in COX-deficient fibres than in COX-positive fibres (Patient 1: COX-deficient fibres: 95.1% ±0.45 (*n*=18), COX-positive fibres: 83.8%±3.38 (*n*=22), *P*=0.005; Patient 2: COX-deficient fibres: 98.0%±0.46 (*n*=20), COX-positive fibres: 78.9%±4.81 (*n*=21), *P*=0.0002; Figures 2c and d), whilst the muscle from both patients showed dramatically decreased mt-tRNA^Ala^ steady-state levels compared with normal controls (Figure 2e).

## Discussion

Histopathological findings of diagnostic muscle biopsies in both patients were characterised by numerous COX-deficient fibres and evidence of subsarcolemmal mitochondrial accumulation. This prompted us to thoroughly investigate the mitochondrial genome. The two novel heteroplasmic mt-tRNA point mutations identified, m.5631G>A and m.5610G>A, are both located in mt-tRNA^Ala^ and are unequivocally pathogenic according to accepted criteria published by Yarham and colleagues.^13^

Additional samples from maternally related members of both patients were available for investigation (Figures 2a and b). Interestingly, one daughter of Patient 2 had obviously higher mutation loads in blood and buccal cells than all other investigated family members, although well below the expected disease threshold for the m.5610G>A mutation. She is healthy, with no sign of muscle weakness although she declined detailed investigations such as assessment of CK levels and formal clinical examination.

mtDNA mutations are frequently associated with multisystemic diseases, although isolated organ involvement is rarely observed. Isolated myopathy owing to mt-tRNA mutations is a rare clinical presentation. Mutations were detected not only in the mt-tRNA^Ala^ gene but single cases are described with mutations in the mt-tRNA^Asp^, mt-tRNA^Trp^, mt-tRNA^Phe^, mt-tRNA^Gln^, mt-tRNA^Leu(CUN)^, mt-tRNA^Pro^, mt-tRNA^Ile^, mt-tRNA^Ser(UCN)^ and mt-tRNA^Lys^ genes. Mutations in the mt-tRNA^Ala^ gene all appear to be associated with isolated myopathy in contrast to mutations in these other mt-tRNA genes that are associated with different phenotypes including multisystemic presentations. In addition to the novel mutations presented here, there are four other previously reported examples of mt-tRNA^Ala^ mutations, two of which are associated with myopathy affecting the limbs (m.5591G>A and m.5650G>A) and two mutations (m.5628T>C and m.5636T>C) associated with a myopathy affecting additionally the extraocular and pharyngeal muscles (Table 1 and Figure 1c). The patient previously described with a m.5591G>A transition presented with pure myopathy, involving predominantly limb girdle muscles, myalgia, elevated CK levels and a severe combined respiratory chain enzyme defect.^14^ Follow-up analysis of urine samples from his two unaffected brothers revealed the presence of the m.5591G>A mutation (67% mutant load) in the younger brother, but not in the older brother.^14^ Initially, the younger brother presented with asymptomatic elevation of CK, later developing exercise-induced muscle weakness leading to proximal paresis at the age of 51 years. The patient reported with a maternally inherited m.5650G>A mutation in mt-tRNA^Ala^ suffered from an increasingly severe limb girdle myopathy.^15^ Two other reported cases showed a preference for skeletal muscle involvement; the m.5628T>C mutation was identified in a patient who presented with proximal paresis, episodic diplopia, external ophthalmoplegia and dysphagia,^16^ whilst the m.5636T>C mutation was identified in a patient who presented with fatigue, bilateral ptosis, external ophthalmoplegia and mild dysarthria.^17^

It is interesting that the threshold for impairment of oxidative phosphorylation, as evidenced by COX deficiency, appears to be extremely high for both novel mt-tRNA^Ala^ mutations. The previously described cases of mt-tRNA^Ala^ mutations associated with a pure myopathic phenotype (m.5591G>A and m.5650G>A) demonstrated similarly high-mutation thresholds, inferring at least a genotype: phenotype correlation for mutations within this particular mt-tRNA gene.

## Figures and Tables

**Table 1:** Clinical, histochemical and molecular details of the novel mt-tRNA^Ala^ mutations and previously reported mt-tRNA^Ala^ mutations

*Mutation (patient)*	*Age of onset (gender)*	*Phenotype*	*Histological findings*	*Level of heteroplasmy in muscle*	*Threshold single fibre level*	*CK (normal: <2.4 μmol/l)*
*m.5631G>A (Patient 1)*	29 (F)	Distal and proximal paresis. Mild symmetrical ptosis, but no external ophthalmoplegia	Numerous COX-deficient fibres (33%) in addition to ragged-red-fibres (2 %)	92%	~96%	Up to 3.9 *μ*mol/l
*m.5610G>A (Patient 2)*	69 (F)	Proximal paresis. No ptosis and no external ophthalmoplegia	Mild myopathic changes. Numerous COX-deficient fibres (40%) in addition to ragged-red-fibres (5%)	91%	~97%	Up to 2.7 *μ*mol/l
*m.5591G>A*^14^	45 (M)	Proximal paresis of arms and legs and distal paresis of both arms. No ptosis and no external ophthalmoplegia	Mild myopathic changes. Marked number (~80%) of COX-deficient fibres with ragged-red fibres (30%)	98%	~98%	Up to 12.31 *μ*mol/l
*m.5591G>*A^14^^a^	51 (M)	Proximal paresis. No ptosis and no external ophthalmoplegia	Numerous number of COX-deficient fibres (60%). Small number of ragged-red fibres	Not determined in muscle; urine: 67%	Not determined	Up to 7.07 *μ*mol/l
*m.5650G>A*^15^	6 (F)	Proximal paresis. Mild bilateral ptosis, but no external ophthalmoplegia	Marked number of COX-deficient fibres (>50%), marked variation in fibre size, evidence of inflammatory changes	>95%	~99%	Up to 6.25 *μ*mol/l
*m.5628T>C*^16^	77 (F)	Proximal paresis, episodic diplopia, external ophthalmoplegia and dysphagia. No ptosis	Many COX-deficient fibres (30%) and ragged-red fibres	40%	Not determined	Normal
*m.5636T>C*^17^	7 (M)	Fatigue syndrome, bilateral ptosis, external ophthalmoplegia, mild dysarthria. No paresis.	Signs of chronic myopathy. Several COX-deficient fibres and ragged-red fibres	31%	>80%	Up to 8.95 *μ*mol/l

a Brother of the patient above who initially presented with asymptomatic elevation of CK levels.
